# Plasma heat shock protein 90alpha as a biomarker for the diagnosis of liver cancer: in patients with different clinicopathologic characteristics

**DOI:** 10.1186/s12957-021-02269-4

**Published:** 2021-08-04

**Authors:** Yueting Han, Youqin Zhang, Lin Cui, Ze Li, Honglei Feng, Ying Zhang, Da Sun, Li Ren

**Affiliations:** 1 Department of Clinical Laboratory, Tianjin Medical University Cancer Institute and Hospital, National Clinical Research Center for Cancer, Tianjin’s Clinical Research Center for Cancer, Key Laboratory of Cancer Prevention and Therapy, Tianjin, 300060 China; 2 Shanghai Institute of Immunology, Department of Immunology and Microbiology, Shanghai Jiao Tong University School of Medicine, Shanghai, 200025 China; 3 Xiangshengtong (Tianjin) Medical Laboratory, Tianjin, 300384 China

**Keywords:** Heat shock protein 90α, Liver cancer, Pathology, Biomarker

## Abstract

**Purposes:**

The purposes of this study were to assess the correlation between the plasma level of Hsp90α and the clinicopathological characteristics of patients with liver cancer and compare the diagnostic efficacy of Hsp90α, AFP, CEA, and CA199 in HCC.

**Experimental design:**

A total of 200 individuals, including 140 patients with liver cancer or benign liver diseases and 60 healthy people, were enrolled for quantitative measurement of plasma Hsp90α by ELISA.

**Results:**

The plasma level of Hsp90α was significantly different between patients with liver cancer or benign liver diseases and healthy controls (*P* < 0.001). The sensitivity, specificity, and AUC (95% CI) of Hsp90α were 93.2%, 85.4%, and 0.931% (0.891–0.972%), respectively, when Hsp90α was applied to differentiate liver cancer patients and healthy controls. Significant positive correlations between the plasma Hsp90α level and clinicopathological characteristics such as the history of basic liver disease (*P* = 0.038), active stage of hepatitis (*P* = 0.039), Child-Pugh score (*P* < 0.001), size of focal liver lesions (*P* = 0.004), and extrahepatic metastasis (*P* < 0.001) were observed. AFP + Hsp90α was the best combination strategy for the auxiliary diagnosis of HCC, with a sensitivity of 95.7%, a specificity of 97.5%, and an AUC of 0.990 (0.976–1.000). The level of plasma Hsp90α decreased significantly (*P* < 0.001) after resection of tumor tissue.

**Conclusions:**

This study demonstrated that plasma Hsp90α levels are useful as a diagnostic biomarker in liver cancer and may predict the responses of patients with liver cancer to surgery. Some clinicopathological characteristics could affect the plasma Hsp90α levels.

## Introduction

Primary liver cancer (PLC) ranks fourth among common malignant tumors. It is the second leading cause of cancer deaths in China. The crude incidence and mortality rates are 26.92/10^5^ and 23.72/10^5^ [1], respectively. Early diagnosis is crucial to the treatment because of the poor prognosis of advanced liver cancer [2, 3]. Good tumor markers play a key role in high-risk screening, early diagnosis, and guiding treatment. Alpha-fetoprotein (AFP) is the most commonly used tumor marker in the screening and clinical diagnosis of patients with liver cancer. The sensitivity and specificity of AFP to hepatocellular carcinoma (HCC) in all stages are 41–65% and 80–94%, respectively [4]. The sensitivity of AFP to HCC in phase A of Barcelona clinical liver cancer is less than 50% [5]. Meanwhile, AFP is less sensitive to intrahepatic cholangiocarcinoma (ICC), which further highlights its incapability in diagnosing different types of liver cancer [6]. Therefore, a new index was needed to complement AFP in clinical practice so as to improve the early diagnostic rate, therapeutic effect, and prognosis of liver cancer.

Heat shock protein 90α (Hsp90α), a conserved and essential molecular chaperone, is important in enabling replicative immortality, inducing angiogenesis, and activating invasion/metastasis of malignant cells. Some studies showed that Hsp90α could be actively translocated into the extracellular space by malignant tumor cells [7]. In addition, the level of Hsp90α in the plasma of patients with malignant tumors increased significantly and correlated positively with the degree of malignancy and the ability of metastasis [8]. A large-scale, multicenter clinical trial data showed that plasma Hsp90α could be used as an early diagnostic marker in the diagnosis of patients with liver cancer, and its diagnostic performance was better than that of AFP (sensitivity 92.7% and specificity 91.3%). In patients undergoing liver surgery or interventional treatment, the dynamic change in the Hsp90α level could help monitor the therapeutic effect [9].

Few studies reported the role of Hsp90α in the patients of liver cancer with different clinicopathologic characteristics, the differentiation of benign and malignant liver diseases, and the monitoring of the curative effect of liver cancer [9, 10]. Although the benefits of HSP90α as a diagnostic factor have been described elsewhere already, we still want to evaluate this biomarker in patients of our center. It is necessary to provide more real-world data, considering the preclinical profile of HSP90α-related solutions, based on the current situation that it lacks previous studies and available cohorts are relatively small. This study enrolled patients with liver cancer admitted to the Tianjin Medical University Cancer Institute and Hospital to explore the role of Hsp90α in liver cancer diagnosis and treatment, providing a scientific basis for its application in this field.

## Methods

### Participants

One-hundred forty treatment-naïve patients who were hospitalized in the Tianjin Medical University Cancer Institute and Hospital from September 2018 to February 2019 for liver diseases, were enrolled in this study. To be specific, 118 patients with liver cancer and 22 patients with liver abscess and hemangioma were, respectively, grouped into liver cancer (postoperative followed-up the patients undergoing hepatic carcinectomy for 8 weeks) and liver benign tumor groups. The staging of these liver cancers was performed according to American Joint Committee on Cancer Classification (AJCC, 8th edition) [11]. In addition, 60 healthy people were included in the control group after the health examination.

The study was approved by the institutional ethics review committee of Tianjin Medical University Cancer Institute and Hospital. Signed informed consent forms were obtained from all participants based on each committee’s regulations.

### Testing of blood samples

Peripheral blood samples (EDTA-K2 anticoagulant) from all participants were collected before anticancer therapy and centrifuged at 3000 rpm for 10 min in 4 h. The plasma was stored at – 20 °C until further use. The plasma Hsp90α concentration was quantitatively measured using a commercially available enzyme-linked immunosorbent assay (ELISA) kit (Batch No. 190126, Protgen Co., Ltd., Yantai, Shandong Province, China) according to the manufacturer’s recommendations. Briefly, diluted plasma samples and standard samples were added to a 96-well microplate precoated with monoclonal antibody to Hsp90α. An HRP-conjugated anti-Hsp90α antibody (50 μL) was added to the plate, and then the plate was incubated at 37 °C for 1 h. The reaction was visualized by adding 50 μL chromogen TMB (solution A) and 50 μL chromogen TMB (solution B) sequentially to each well and incubated for 20 min at 37 °C. Finally, the reaction was stopped by adding 50 μL Stop Solution to each well. The optical density was measured at 450 nm and referenced to 570 nm on a spectrophotometer. The standard curve was generated by plotting the logarithm of average optical density obtained for each of the six standard samples on the vertical (*Y*) axis versus the logarithm of corresponding concentrations on the horizontal (*X*) axis. The absorbance of samples was then calculated with the method of substitution in the standard curve to determine the level of Hsp90α in the plasma sample. Double logarithmic curve fitting was recommended, and the coefficient of correlation (*R*^2^) was required to be more than 0.980.

The serum levels of carcinoembryonic antigen (CEA), AFP, and CYFRA21-1 were also measured using a commercially available chemoluminescence (CL) kit (R&D Systems).

### Follow-up

For the patients with liver cancer who continued to receive postoperative follow-up, plasma samples were still collected in the 4th week after surgery to do other laboratory examination (including blood routine test, assessment of liver and kidney function, five items for hepatitis B, hepatitis B virus (HBV) DNA, AFP, and Hsp90α). Besides, the imaging examination (chest, abdomen, pelvic enhancement CT, and/or MRI) was performed at the 8th week, then those results were evaluated and described by “complete response” (CR), “partial response” (PR), “stable disease” (SD), and “progressive disease” (PD) according to “Response Evaluation Criteria in Solid Tumors (RECIST) v1.1” [12].

### Statistical analysis

Statistical analysis was performed using SPSS v21.0 software. The counting data were expressed as a percentage, and the *χ*^2^ test was used for comparison. The measurement data was described as mean ± standard deviation. The one-way analysis of variance test was used for comparing independent samples with a normal distribution, and the rank-sum test was used for comparing independent samples with a nonnormal distribution. A *P* value less than 0.05 was considered statistically significant. Receiver operating characteristic (ROC) curves were constructed to assess the sensitivity, specificity, and respective areas under the curves (AUCs) with a 95% confidence interval (CI). The optimum cutoff value was determined for the diagnosis by maximizing the sum of sensitivity and specificity, minimizing the square root of the sum [(1 − Sensitivity)^2^ + (1 − Specificity)^2^], and minimizing the distance between the point and the top-left corner of the ROC curve (where sensitivity = 1 and specificity = 1). The correlation between the clinicopathological characteristics and the plasma Hsp90α level in the liver cancer group was analyzed using the Spearman rank correlation analysis. The diagnostic efficacy of the combined detection of multiple tumor markers was analyzed using the binary logistic regression analysis

## Results

### Clinical data

This study enrolled 118 patients (average age of 50.69 ± 9.47 years) with liver cancer. At baseline, most of them had history of basic liver disease and were in the advanced stage. Forty-four patients with liver cancer underwent hepatic carcinectomy. Thirty three patients had a recurrence during the follow-up. The clinicopathologic characteristics from patients with liver and lung cancer were obtained and are summarized in Table 1.

**Table 1:** Clinicopathologic characteristics of patients with liver cancer

Characteristics		*Number (percentage)*
Sex (*n* = 118)	Male	87 (74)
	Female	31 (26)
Age (*n* = 118)	< 60	66 (56)
	≥ 60	52 (44)
Source (*n* = 118)	Primary	91 (77)
	Secondary	27 (23)
PLC histologic types (*n* = 91)	HCC	67 (74)
	ICC	20 (22)
	Other types	4 (4)
TNM stage (*n* = 118)	I	20 (17)
	II	12 (10)
	III	27 (23)
	IV	59 (50)
Primary organs of secondary liver cancer (*n* = 27)	Colorectal	10 (37)
	Stomach	3 (11)
	Pancreas	4 (15)
	Other	10 (37)
History of basic liver disease (*n* = 118)	Viral hepatitis	59 (50)
	Liver cirrhosis	39 (33)
	No basic liver disease	20 (17)
Child-Pugh (*n* = 118)	A/B	59 (50)
	C	59 (50)
Morbidity (*n*=118)	Not recurrence	85 (72)
	Recurrence	33 (28)

### Auxiliary diagnostic effect of plasma Hsp90α level on liver cancer

**Fig. 1:**
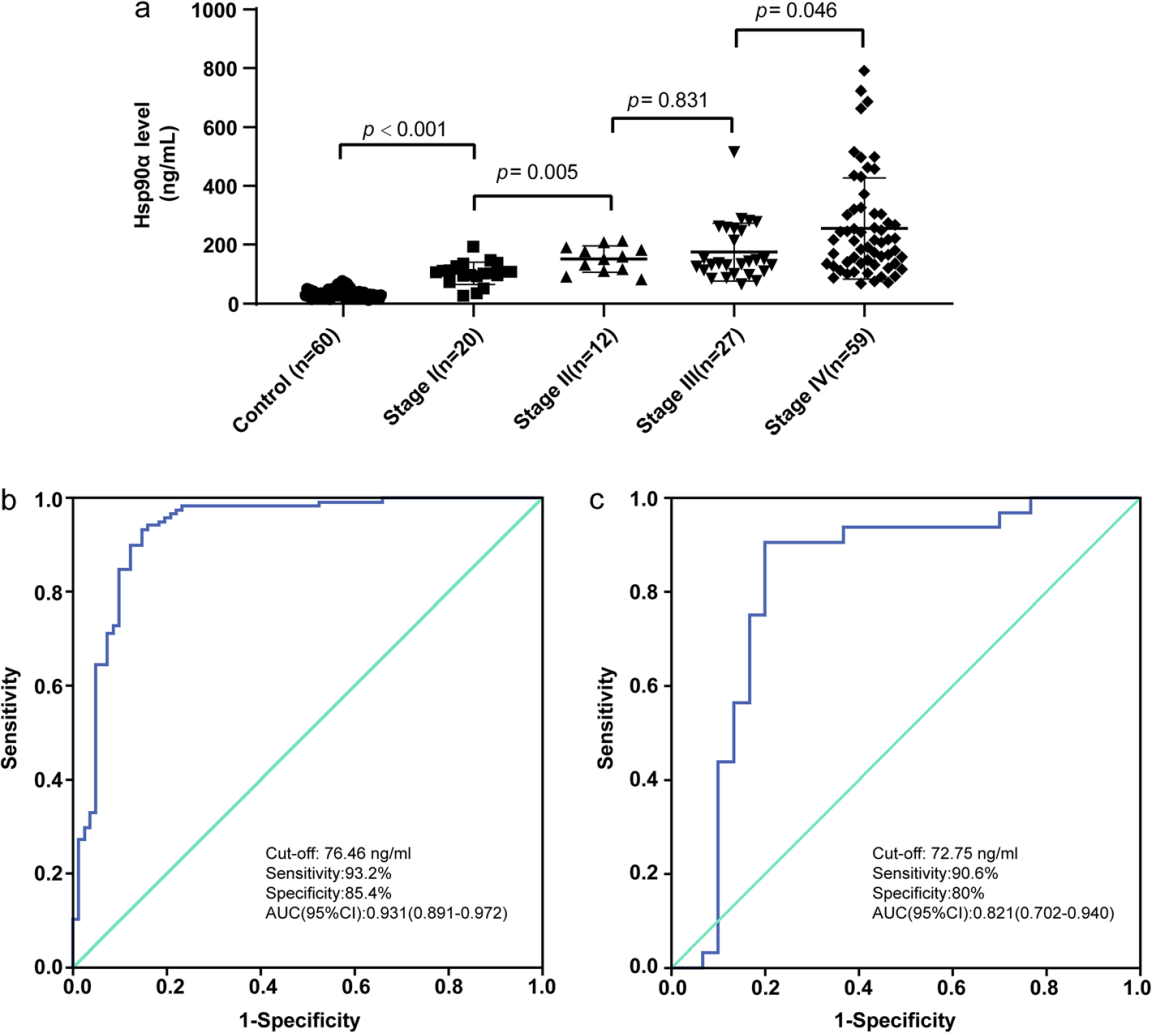
Plasma levels of Hsp90α in patients with different stages of liver cancers and Hsp90α ROC curve of auxiliary diagnosis of liver cancer. **a** shows plasma Hsp90α levels in liver cancer patients with TNM I–IV .Compare the plasma Hsp90α levels of patients in each stage, I and II *(P* = 0.005), III and IV (*P* = 0.046), showed significant differences, II and III (*P* = 0.831) showed no significant differences in plasma Hsp90α levels. **b** shows ROC curve of plasma Hsp90α for I–IV liver cancer patients versus non-liver cancer group. **c** shows ROC curve of plasma Hsp90α for I–II liver cancer patients versus non-liver cancer group. *Hsp90α*, plasma heat shock protein 90 alpha; *LC*, liver cancer; *ROC*, receiver operating characteristic; *AUC*, area under curve

The results showed that the level of Hsp90α in the plasma of the liver cancer group, benign tumor group, and healthy control group was significantly different (*P* < 0.001). The plasma level of Hsp90α was significantly higher in patients with liver cancer (200.58 ± 143.64 ng/mL) than in patients with benign tumors (110.64 ± 90.06 ng/mL) and healthy controls (35.07 ± 15.42 ng/mL). Among these, the plasma level was significantly higher in patients with stage I and stage II liver cancer (resectable) than in those with non-liver cancers (*P* < 0.001). The plasma level of Hsp90α was not statistically significant (*P* = 0.831) in different histological types of primary liver cancer. The plasma level of Hsp90α in patients with different stages of liver cancers was statistically significant (*P* = 0.005, 0.046, and <0.001 for I vs II, III vs IV, and I + II vs III + IV, respectively). According to the ROC curve, the sensitivity, specificity, and AUC (95% CI) were 93.2%, 85.4%, and 0.931% (0.891–0.972%), respectively (see Tables 2 and 3 and Fig. 1).

**Table 2:** Plasma Hsp90α levels in different groups

Group	Subgroup		Number	Plasma Hsp90α level (ng/mL)
Liver cancer	-		118	200.58 ± 143.64
	PLC		91	190.38 ± 125.72
	Types	HCC	67	201.48 ± 152.22
		ICC	20	172.21 ± 89.66
		Other types	4	280.83 ± 49.65
	Stage	I	20	103.42 ± 37.80
		II	12	151.59 ± 44.16
		III	27	174.85 ± 97.65
		IV	59	255.26 ± 171.48
Liver benign disease	-		22	110.64 ± 90.06
Healthy control	-		60	35.07 ± 15.42

**Table 3:** *P* value of the plasma Hsp90α level in different stages of liver cancer

Group	*P* value
I vs II	**0.005**
II vs III	0.831
III vs IV	**0.046**
I + II vs III + IV	**< 0.001**
Non-liver cancer vs I + II	**< 0.001**
I vs III	**0.034**
I vs IV	**0.002**
II vs IV	**0.042**

**Bold** *P* values are significant.

### Plasma levels of Hsp90α in patients with liver cancer with different clinicopathological characteristics

The results showed that the history of basic liver disease; active stage of hepatitis; primary, recurrent, and secondary cancers; extrahepatic metastasis; size of focal liver lesions; and classification of liver function had a significant influence on the plasma level of Hsp90α. Meanwhile, no difference in the plasma level of Hsp90α was found between patients of different ages, sexes, histological sources, and primary organs of secondary liver cancers (see Table 4).

**Table 4:** Plasma levels of Hsp90α in patients with liver cancer with different clinicopathological characteristics

Characteristic		Number	Plasma Hsp90α level (ng/mL)	*P* value
Sex	Male	87	207.79 ± 144.91	0.153
	Female	31	180.35 ± 140.34	
Age	< 60	66	221.23 ± 160.92	0.184
	≥ 60	52	174.38 ± 114.34	
History of hepatitis	Yes	59	245.14 ± 181.37	**0.024**
	No	59	156.02 ± 68.42	
History of cirrhosis	Yes	39	269.71 ± 206.83	**0.040**
	No	79	166.45 ± 80.85	
Active stage of liver disease	Yes^*^	17	221.05 ± 148.62	**0.040**
	No	17	134.07 ± 26.79	
Child-Pugh	A/B	59	155.21 ± 100.03	**0.025**
	C	59	255.26 ± 171.48	
Morbidity	1st Occurrence	118	217.75 ± 104.27	**0.042**
	Recurrence	33	136.12 ± 49.23	
Source	Primary	91	190.38 ± 125.72	**0.038**
	Secondary	27	249.78 ± 154.61	
Primary organs of secondary liver cancer	Colorectal	10	221.90 ± 192.85	0.896
	Stomach	3	133.89 ± 41.05	
	Pancreas	4	205.56 ± 137.48	
	Other	10	220.12 ± 186.96	
Size of focal liver lesions	< 3 cm	10	123.31 ± 26.01	**0.006**
	3 cm	22	177.71 ± 54.33	
Extrahepatic metastasis	Yes	33	277.20 ± 183.01	**< 0.001**
	No	22	115.11 ± 61.26	
	N/A	63	212.24 ± 133.91	

* HBV DNA > 10^4^. **Bold** *P* values are significant.

### Correlation between the plasma level of Hsp90α and the clinicopathological characteristics of patients with liver cancer

The results showed a significantly positive correlation between the plasma Hsp90α level and clinicopathological characteristics such as the history of basic liver disease, active stage of hepatitis, Child-Pugh score, size of focal liver lesions, and extrahepatic metastasis. That is to say, a history of basic liver disease, active stage of basic liver disease, decompensation of liver function, size of focal liver lesions > 3 cm, and extrahepatic metastasis could increase the plasma Hsp90α level (see Table 5).

**Table 5:** Correlation between the plasma level of Hsp90α and the clinicopathological characteristics of patients with liver cancer

Clinicopathological characteristics	Spearman coefficient (*ρ*)	*P* value
History of hepatitis	0.209	**0.038**
History of cirrhosis	0.190	**0.019**
Active stage of liver disease	0.357	**0.039**
Child-Pugh score	0.224	**< 0.001**
First occurrence/recurrence	0.044	0.185
Primary/secondary	0.055	0.266
Size of focal liver lesions	0.493	**0.004**
Extrahepatic metastasis	0.565	**< 0.001**

**Bold** *P* values are significant.

### Diagnosis of HCC by Hsp90α combined with AFP, carcinoembryonic antigen (CEA), and carbohydrate antigen199 (CA199)

**Fig. 2:**
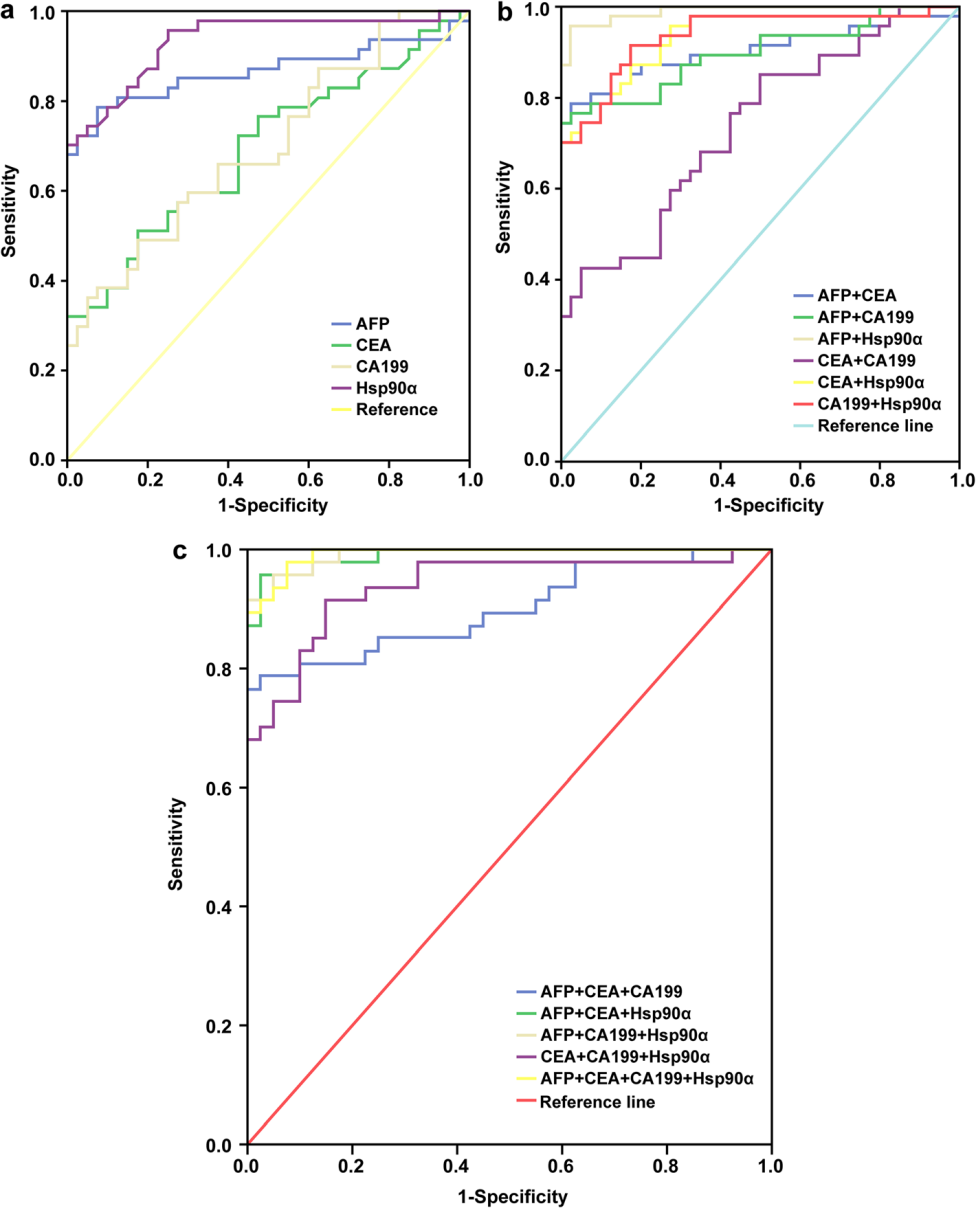
ROC curve of four tumor markers for HCC. **a** shows the ROC curve of Hsp90α, AFP, CEA, and CA-199 alone in HCC patients. **b** shows the ROC curve of the combined application of any two of the four biomarkers Hsp90α, AFP, CEA, and CA-199 in HCC patients. **c** shows the ROC curve of the combination of any three of the four biomarkers Hsp90α, AFP, CEA, and CA-199 in HCC patients. *ROC*, receiver operating characteristic; *HCC*, hepatocellular carcinoma; *SEN*, sensitivity; *SPE*, specificity; *Hsp90α*, plasma heat shock protein 90 alpha; *AFP*, alpha-fetoprotein; *CEA*, carcinoembryonic antigen; *CA199*, carbohydrate antigen199

Among the 67 patients with HCC, 47 patients had AFP, CA199, and CEA baseline data, whereas among the 60 individuals in the control group, 40 patients had AFP, CA199, and CEA test data. The data analysis showed that for the four tumor markers, including Hsp90α, Hsp90α had the highest sensitivity (95.7%), and AFP had the highest specificity (92.5%). The ROC curve of single and combined use of the four indicators showed that AFP + Hsp90α was the best combination strategy for the auxiliary diagnosis of HCC, with a sensitivity of 95.7%, a specificity of 97.5%, and an AUC of 0.990 (95% CI, 0.976–1.000) (see Table 6 and Fig. 2).

**Table 6:** Diagnostic efficacy of four tumor markers in HCC

Index(es)	AUC (95% CI)	Sensitivity (%)	Specificity (%)	PPV (%)	NPV (%)
AFP	0.866 (0.783–0.950)	78.7	**92.5**	78.7	92.5
Hsp90α	0.934 (0.882–0.986)	**95.7**	75.0	**95.7**	75.0
CEA	0.694 (0.584–0.804)	51.1	81.5	27.7	**100**
CA199	0.700 (0.592–0.808)	48.9	81.5	48.9	85.0
AFP + Hsp90α	0.990 (0.976–1.000)	**95.7**	97.5	**74.5**	70.0
AFP + CA199	0.896 (0.829–0.964)	74.5	100	40.4	72.5
AFP + CEA	0.903 (0.933–0.972)	78.7	97.5	23.4	**75.0**
CEA + Hsp90α	0.932 (0.879–0.984)	70.2	**100**	27.7	60.0
CA199 + Hsp90α	0.936 (0.885–0.987)	91.5	81.5	48.9	62.5
CEA + CA199	0.736 (0.633–0.839)	42.6	95.0	25.5	70.0
AFP + CEA + CA199	0.900 (0.833–0.967)	76.6	100	21.3	**62.5**
AFP + CEA + Hsp90α	0.990 (0.976–1.000)	**95.7**	97.5	23.4	57.5
AFP + CA199 + Hsp90α	0.991 (0.980–1.000)	91.5	**100**	**40.4**	57.5
CEA + CA199 + Hsp90α	0.938 (0.888–0.989)	91.5	85.0	25.5	55.0
Four indexes combined	0.993 (0.982–1.000)	97.9	92.5	21.3	50.0

### Dynamic changes of plasma levels of Hsp90α pre- and post-hepatic carcinectomy

Dynamic changes reflecting the patients’ condition could provide the clinical guidance for doctors. We therefore tentatively explored the efficacy monitoring capability of plasma Hsp90α in patients undergoing hepatic carcinectomy. The results showed that the level of plasma Hsp90α decreased significantly (*P* < 0.001) after resection of tumor tissue in 44 patients from 249.29 ± 142.82 to 131.01 ± 63.23 ng/mL (see Table 7).

**Table 7:** Plasma levels of Hsp90α pre- and post-hepatic carcinectomy

	Number	Plasma Hsp90α level (ng/mL)	*P* value
Preoperative	44	249.29 ± 142.82	< 0.001
Postoperative		131.01 ± 63.23	

## Discussion

Hsp90α is a molecular chaperone involved in a variety of physiological and pathological signaling pathways. It is overexpressed in a variety of tumors, such as liver cancer, colorectal cancer, gastric cancer, lung cancer, breast cancer [8, 13–17], and so on. Similar to the result of a former large-scale, multicenter clinical trial, we found that plasma Hsp90α exhibited a significantly higher diagnostic performance for liver cancer than AFP [9]. The results of the present study also confirmed that Hsp90α could assist AFP in liver cancer diagnosis by improving the sensitivity and specificity of HCC. Compared with single detection, the combination of the two could further improve the sensitivity to 95.7% and specificity to 97.5%. Therefore, the diagnosis using a combination of Hsp90α and AFP is more economical and appropriate in clinical practice.

Transcriptome–proteome integrated assay showed that Hsp90α is overexpressed in the HCC cells, serum, and tissues, and related to CC metastatic behavior and cancer-related signaling pathways [18]. The plasma level of Hsp90α in our patients with liver cancer showed a significant upward trend with the progression in the clinical stage, and a significant positive correlation was observed. In particular, the level of Hsp90α was significantly lower in patients with stage I and stage II liver cancers than in those with stage III and stage IV liver cancers, and significantly higher than in patients with benign liver disease. It meant (1) Hsp90α could reflect the malignant degree and disease progression of liver cancer and provided evidence for treatment plans. For example, most stages I–II liver cancers could be treated locally to achieve the goal of radical cure, indicating that Hsp90α had the potential to indicate whether it was feasible to receive the treatment of operation or ablation. (2) Hsp90α has the potential to detect early-stage or small liver cancer, which could not be detected by current imaging approaches. Hence, it could play a certain role in the screening and follow-up of the high-risk population to improve the early diagnostic rate and resectability rate of liver cancer, or assist an imaging examination to detect small residual, recurrent, or metastatic lesions. It could also improve the prognosis and prolong the survival period of patients. Relevant basic research showed that Hsp90α was secreted out of cells and promoted the invasion and metastasis of tumor cells by combining with the important invasion-related factor matrix metalloproteinase-2 [7, 19]. In the early stage of tumor evolution, the preparation work of distant metastasis had begun [20].

The clinical characteristics of patients with liver cancer, including history of basic liver disease, active stage of basic liver disease, and different liver function grades, significantly affect the plasma level of Hsp90α. This indicates that the clinical application of Hsp90α in the auxiliary diagnosis or differential diagnosis of liver cancer requires to pay close attention to the clinical condition and other related laboratory tests, so as to avoid false-positive results and overdiagnosis and treatment as much as possible. Especially in the early diagnosis, the examination of the related symptoms and signs, drinking history, related basic liver disease and treatment history, HBV DNA test results, biochemical indicators of liver function (especially ALT/AST ratio), and so on are very important to the correct interpretation of Hsp90α. Correspondingly, data from a published clinical trial ever indicated the outstanding performance of Hsp90α in diagnosing early cancer [21]. Meanwhile, it was speculated that the level of plasma Hsp90α in patients with liver cancer who were diagnosed and treated could be used as a potential indicator of long-term prognosis. The synthesis and secretion of Hsp90α by malignant tumor cells are independent to some extent, but the state of liver function is also an important factor. Therefore, poor antiviral effect, decompensation of liver function, large primary liver cancer, and late clinical stage are not only the factors for Hsp90α overexpression but also the adverse factors for liver cancer prognosis [2, 22–26].

Furthermore, we found that plasma Hsp90α fluctuance could reflect patient’s response to hepatic carcinectomy, as there was a significant difference between preoperative and postoperative Hsp90α levels. Plasma levels of Hsp90α appeared to be associated with decreased tumor burden by surgery, and that was consistent with the existing reports [7]. It hints us that Hsp90α is a good monitor for patients undergoing hepatic carcinectomy.

There is no doubt that some limitations in the present study should be realized. First, our study was a single-center trial, and our patients were retrospectively enrolled. Our results should be further evaluated in large-sample prospective studies. Second, patients enrolled may not be able to represent the general population with liver cancer due to the relatively small sample size. We could not exclude the possibility of disease spectrum bias. Last but not least, we did not touch upon liver cancers related to nonalcoholic fatty liver disease (NAFLD) and alcohol-associated liver disease (AALD).

## Conclusion

To sum up, plasma Hsp90α could serve as a useful diagnostic biomarker in liver cancer as well as a predictor for surgery response. As it associates with several clinicopathological characteristics, further basic and clinical researches should be carried to elucidate the mechanisms regarding its fluctuance in patients and to explore novel therapeutic approaches.

## Data Availability

Not applicable

## References

[1] Sun K, Zheng R, Zeng H (2019). Report of cancer incidence and mortality in different areas of China. 2015. China Cancer.

[2] National Health and Family Planning Commission of PRC (2017). Standardization of diagnosis and treatment for hepatocellular carcinoma (2017 edition). Chin J Digest Surg.

[3] Cillo U, Vitale A, Grigoletto F, Farinati F, Brolese A, Zanus G (2006). Prospective validation of the Barcelona Clinic Liver Cancer staging system. J Hepatol.

[4] Gupta S, Bent S, Kohlwes J (2003). Test characteristics of alpha-fetoprotein for detecting hepatocellular carcinoma in patients with hepatitis C. A systematic review and critical analysis. Ann Intern Med.

[5] Singal AG, Conjeevaram HS, Volk ML, Fu S, Fontana RJ, Askari F (2012). Effectiveness of hepatocellular carcinoma surveillance in patients with cirrhosis. Cancer Epidemiol Biomark Prev.

[6] Patel T (2001). Increasing incidence and mortality of primary intrahepatic cholangiocarcinoma in the United States. Hepatology..

[7] Eustace BK, Sakurai T, Stewart JK, Yimlamai D, Unger C, Zehetmeier C (2004). Functional proteomic screens reveal an essential extracellular role for Hsp90 alpha in cancer cell invasiveness. Nat Cell Biol.

[8] Wang X, Song X, Zhuo W, Fu Y, Shi H, Liang Y (2009). The regulatory mechanism of Hsp90alpha secretion and its function in tumor malignancy. Proc Natl Acad Sci U S A.

[9] Fu Y, Xu X, Huang D, Cui D, Liu L, Liu J (2017). Plasma heat shock protein 90alpha as a biomarker for the diagnosis of liver cancer: an official, large-scale, and multicenter clinical trial. EBioMedicine..

[10] Liu W, Li J, Zhang P, Hou Q, Feng S, Liu L (2019). A novel pan-cancer biomarker plasma heat shock protein 90alpha and its diagnosis determinants in clinic. Cancer Sci.

[11] Amin B, Edge SB, Greene L (2017). AJCC cancer staging manual.

[12] Eisenhauer EA, Therasse P, Bogaerts J, Schwartz LH, Sargent D, Ford R (2009). New response evaluation criteria in solid tumours: revised RECIST guideline (version 1.1). Eur J Cancer.

[13] Chen JS, Hsu YM, Chen CC, Chen LL, Lee CC, Huang TS (2010). Secreted heat shock protein 90alpha induces colorectal cancer cell invasion through CD91/LRP-1 and NF-kappaB-mediated integrin alphaV expression. J Biol Chem.

[14] Ding J, He X, Cheng X, Cao G, Chen B, Chen S (2021). A 4-gene-based hypoxia signature is associated with tumor immune microenvironment and predicts the prognosis of pancreatic cancer patients. World J Surg Oncol.

[15] Shi Y, Liu X, Lou J, Han X, Zhang L, Wang Q (2014). Plasma levels of heat shock protein 90 alpha associated with lung cancer development and treatment responses. Clin Cancer Res.

[16] Tian WL, He F, Fu X, Lin JT, Tang P, Huang YM (2014). High expression of heat shock protein 90 alpha and its significance in human acute leukemia cells. Gene..

[17] Yang H, Huo J, Li X (2021). Identification and validation of a five-gene prognostic signature for hepatocellular carcinoma. World J Surg Oncol.

[18] Zhou Y, Deng X, Zang N, Li H, Li G, Li C (2015). Transcriptomic and proteomic investigation of Hsp90A as a potential biomarker for HCC. Med Sci Monit.

[19] Eustace BK, Jay DG (2004). Extracellular roles for the molecular chaperone, Hsp90. Cell Cycle.

[20] Harper KL, Sosa MS, Entenberg D, Hosseini H, Cheung JF, Nobre R (2016). Mechanism of early dissemination and metastasis in Her2(+) mammary cancer. Nature..

[21] Kasanga M, Liu L, Xue L, Song X (2018). Plasma heat shock protein 90-alpha have an advantage in diagnosis of colorectal cancer at early stage. Biomark Med.

[22] Budhu A, Forgues M, Ye QH, Jia HL, He P, Zanetti KA (2006). Prediction of venous metastases, recurrence, and prognosis in hepatocellular carcinoma based on a unique immune response signature of the liver microenvironment. Cancer Cell.

[23] Hao K, Luk J, Lee P (2009). Predicting prognostics in hepatocellular carcinoma after curative surgery with common clinicopathologic parameters. BMC Cancer.

[24] Kim JH, Choi MS, Lee H, Kim DY, Lee JH, Koh KC (2006). Clinical features and prognosis of hepatocellular carcinoma in young patients from a hepatitis B-endemic area. J Gastroenterol Hepatol.

[25] Poon RT, Fan ST, Lo CM, Liu CL, Wong J (1999). Intrahepatic recurrence after curative resection of hepatocellular carcinoma: long-term results of treatment and prognostic factors. Ann Surg.

[26] Yin J, Lin N, Han Y (2013). Effect of antiviral treatment with nucleotide/nucleoside analogs on postoperative prognosis of hepatitis B virus-related hepatocellular carcinoma: a two-stage longitudinal clinical study. J Clin Oncol.

